# Effect of Inulin on Proteome Changes Induced by Pathogenic Lipopolysaccharide in Human Colon

**DOI:** 10.1371/journal.pone.0169481

**Published:** 2017-01-09

**Authors:** Michele Pier Luca Guarino, Annamaria Altomare, Simone Barera, Vittoria Locato, Silvia Cocca, Cinzia Franchin, Giorgio Arrigoni, Candida Vannini, Sarah Grossi, Paola Campomenosi, Valentina Pasqualetti, Marcella Bracale, Rossana Alloni, Laura De Gara, Michele Cicala

**Affiliations:** 1 Gastroenterology Unit, University Campus Bio-Medico di Roma, Rome, Italy; 2 Department of Biotechnology and Life Science, University of Insubria, Varese, Italy; 3 Food Sciences and Human Nutrition Unit, University Campus Bio-Medico di Roma, Rome, Italy; 4 Department of Biomedical Sciences, University of Padova, Padova, Italy; 5 Proteomics Center, University of Padova and Azienda Ospedaliera di Padova, Padova, Italy; 6 Surgery Unit, University Campus Bio-Medico di Roma, Roma, Italy; National Institute for Agronomic Research, FRANCE

## Abstract

In the present study, the protective role of inulin against lipopolysaccharide (LPS)-induced oxidative stress was evaluated on human colonic mucosa using a proteomic approach. Human colonic mucosa and submucosa were sealed between two chambers, with the luminal side facing upwards and overlaid with Krebs (control), LPS or LPS+ inulin IQ solution. The solutions on the submucosal side (undernatants) were collected following 30 min of mucosal exposure. iTRAQ based analysis was used to analyze the total soluble proteomes from human colonic mucosa and submucosa treated with different undernatants. Human colonic muscle strips were exposed to the undernatants to evaluate the response to acetylcholine. Inulin exposure was able to counteract, in human colonic mucosa, the LPS-dependent alteration of some proteins involved in the intestinal contraction (myosin light chain kinase (MLCK), myosin regulatory subunit (MYL)), to reduce the up-regulation of two proteins involved in the radical-mediated oxidative stress (the DNA-apurinic or apyrimidinic site) lyase) APEX1 and the T-complex protein 1 subunit eta (CCT7) and to entail a higher level of some detoxification enzymes (the metallothionein-2 MT2A, the glutathione–S-transferase K GSTk, and two UDP- glucuronosyltransferases UGT2B4, UGT2B17). Inulin exposure was also able to prevent the LPS-dependent intestinal muscle strips contraction impairment and the mucosa glutathione level alterations. Exposure of colonic mucosa to inulin seems to prevent LPS-induced alteration in expression of some key proteins, which promote intestinal motility and inflammation, reducing the radical-mediated oxidative stress.

## Introduction

Fructans, such as inulin, are dietary fibers, which stimulate gastro-intestinal function by acting as prebiotics. They are characterized by resistance to digestion, fermentability and selectivity in promoting the growth or activity of beneficial bacteria [1]. Resistance to small-intestinal digestion is due to the lack of enzymes that hydrolyze the polymer bonds in humans. This allows the prebiotic to reach the colon intact and undergo fermentation by a limited number of bacteria genera/species. Interestingly, we have recently demonstrated that inulin preserves its antioxidant capability following cooking and simulated digestion processes [2].

The interaction between dietary intake and the microbiota in healthy people has been recognized for many years. However, evidence of the interaction between prebiotics, gastro-intestinal (GI) microbiota and digestive disorders is now emerging, in part due to the development of more robust approaches to examine dietary intake, complex microbial ecosystems and disease outcomes [3]. In animal models, prebiotics have been reported to provide beneficial effects either by increasing fecal IgA levels [4] or by directly modulating host cell gene responses [5], and it has been demonstrated that prebiotics can modulate both adaptive and innate immune systems both in animals and in humans [6,7]). Inulin represents the most commonly used prebiotic and it has been demonstrated that, in co-administration with probiotics, it promotes probiotic-induced anti-inflammatory effects [8,9]. It was shown that combination of inulin with *Lactobacillus plantarum* LS/07 CCM7766 abolishes 1,2-dimethylhydrazine (DMH)-induced inflammatory process in the jejunal mucosa by inhibiting the production of pro-inflammatory cytokines and inducible nitric oxide synthase (iNOS) and by stimulation of anti-inflammatory cytokine synthesis [10].

We have recently demonstrated the protective effect of inulin on lipopolysaccharide (LPS)-induced damage of colonic smooth muscle in an *ex vivo* experimental model, which seems to be related to the presence of oxidative stress [2]. LPS is known to be a potential mediator of multisystem organ failure; it has been shown that endotoxemia results in a significant impairment of intestinal smooth muscle contractility in animal models [11] can alter the kinetic properties of human colonic muscle cell (SMCs) [12,13]. These effects seem to be related to activation of muscular macrophages by mucosal translocation of LPS, which can bind to specific receptor on SMCs, or by mucosal oxidative stress; activated muscular macrophages then secrete several mediators including prostaglandins, H_2_O_2_, cytokines and nitric oxide [12–15].

For this reason, LPS represents a good model for studying functional GI disorders. The beneficial effect of inulin on LPS-induced muscle cell impairment, that we observed in our previous work, could therefore be related to the ability to counteract the oxidative damage induced by LPS in the colonic mucosa since the level of protein oxidation induced by LPS exposure was remarkably reduced when the tissue was treated with inulin [2]. However, little is known about the specific mechanisms by which inulin acts on intestinal muscle function and the molecular mechanisms involved in the direct and/or indirect response of colonic mucosa to this prebiotic. In the present study, the protective role of inulin against oxidative stress induced by LPS in colonic mucosa was evaluated using the Isobaric Tags for Relative and Absolute Quantification (iTRAQ) proteomic approach. iTRAQ is one of the most reliable methods to relatively and absolutely quantify proteins. This mass spectrometry-based technique enables the unbiased evaluation of protein expression in complex biological samples and has wide application in the biological and biomedical sciences [16]. Furthermore, since inulin does not cross the plasma membrane of colonic epithelial cells, we expected that the protective role against cellular protein oxidation could be mediated by a direct inulin effect on cellular redox state. Finally, we evaluated the effect of the prebiotic on colonic muscle strips, which, compared to isolated muscle cells, better represent the functional activity of the intestine.

## Materials and Methods

The experimental protocols were approved by the Ethics Committee of Campus Bio-Medico University of Rome (Prot 20.09 ComEt CBM). Written informed consent was obtained from all patients prior to surgery.

### Chemicals

For the experiments reported in this study inulin Fructafit IQ Instant Quality, (Sensus, Roosendal, The Netherlands) from chicory roots was used. This inulin consists of linear polymers with degree of polymerization of 8–13. For the purpose of this study it was highly purified (90–99.5%) with a residual contamination of fructose, sucrose or glucose. All the other chemicals were purchased from Sigma-Aldrich, Milan, Italy.

For the proteomic analysis, the iTRAQ 4-plex reagents were purchased from AB Sciex (Framingham, MA, USA) and all other reagents were of MS grade.

### Tissue Specimens

Normal colonic mucosa was obtained from the healthy margins of cancer resections from six patients with adenocarcinoma of the colon (M:F = 4:2, age range 48–73 years), treated at the Campus Bio-Medico University of Rome (Italy) between September 2014 and November 2015. None of these patients had a history of colonic motility or a neuromuscular or collagen disorder; specimens with *diverticula* were excluded. Left hemi-colectomy was performed in all patients, affected by carcinoma of the sigma and a specimen from the transverse colon margin was obtained, to be sure that tissue used for the experimental set up was at a distance from the area involved by the carcinoma. Fresh specimens were brought to the laboratory in chilled Krebs solution (containing (in mM) 116.6 mM NaCl, 21.9 mM NaHCO_3_, 1.2 mM KH_2_PO_4_, 5.4 mM glucose, 1.2 mM MgCl_2_, 3.4 mM KCl, and 2.5 mM CaCl_2_), gassed with carbogen (95% O_2_ and 5% CO_2_).

### Experimental Set-Up

After removal of the muscle layer and serosa, the tissue containing mucosa and submucosa was sealed between two tubes with the luminal side of the mucosa facing upwards and submucosal side facing downwards, as previously described [13]. Inflammation conditions were induced by LPS from *Escherichia coli* pathogenic strain serotype O111:B4 (Sigma-Aldrich, Milan, Italy) exposure.

In the experimental model, the luminal side of the mucosa was overlaid with 5 mL of Krebs (N-supernatant), 5 mL of 100 μg/mL LPS solution (500,000 endotoxin units/mg) in Krebs (LPS-supernatant), or with 5 mL of LPS + 100 mg/mL inulin IQ solution in Krebs (LPS+INU-supernatant). LPS concentration was chosen based on previous evidence [2]. The whole biological system was maintained for 30 minutes at 37°C in a thermostatic bath, which was constantly oxygenated. Smaller and larger tubes were adopted to guarantee that the set-up was well sealed; the efficacy of this operation was ensured by monitoring the level of the solution in the smaller tube [2].

After 30 minutes, the Krebs solutions on the submucosal side, in the absence (N-undernatant) or presence of LPS (LPS-undernatant) and LPS-inulin (LPS+INU-undernatant), were collected to evaluate their effects on smooth muscle strips (SMSs) contractility (see below). As previously demonstrated by histopathological evaluation [13], in this experimental set up the dissected mucosa and submucosa do not show necrotic signs or significant inflammatory infiltrates in the lamina propria or in the submucosal layer before and after 30 minutes exposure to undernatants. Finally, human colonic mucosa was stored at -80°C in order to perform proteomic analysis.

### Total Protein Extraction

50 mg of each sample (six patients, three conditions per patient: N-, LPS-, LPS+INU- treated mucosa samples) were disrupted in liquid nitrogen and suspended in 1 mL of 6 M Urea, 2 mM Thiourea, 40 mM Tris pH, 0.1% w/v CHAPS, 0.1% w/v DTT, supplemented with protease inhibitors (0.5 mM phenylmethylsulfonyl fluoride, 1mM benzamidine, and 1 μM leupeptin). The homogenate was incubated for 2 h and then centrifuged at 10.000g for 30 min at 4°C. The supernatant was collected, diluted 1:5 in 80% acetone and kept at -20° C overnight. The samples were centrifuged at 12.000g for 20 min at 4°C. The resultant pellets were re-suspended in 0.5 M triethylammonium bicarbonate solution with 0.1% SDS and quantified. The protein concentration was determined according to the Bradford method, with eight replicates for each sample. Three independent protein extractions were performed for each condition tested.

### Sample Preparation for iTRAQ Analysis

For each treatment, equal amounts (20μg) of proteins extracted from six patients were pooled. The pools derived from N-, LPS- and LPS+INU- treated mucosal samples were named Control, LPS and LPS+INU respectively.

60 μg of each pool were reduced with 10 mM dithiothreitol (DTT) for 1 h at 56°C, alkylated with 55 mM iodoacetamide for 45 min in the dark, and digested using sequencing grade modified trypsin (Promega) at a ratio of 1:35 (w:w) for 18 h at 37°C. Peptides were labelled using an iTRAQ 4- plex kit (AB Sciex Inc., Framingham, MA, USA) according to the manufacturer’s protocol. The quantitative analysis was carried out on two completely independent replicates and pools were labelled with iTRAQ tags according to the following scheme: for the first replicate 114 for Control, 116 for LPS, and 117 for LPS+INU; for the second replicate, 114 for LPS+INU, 115 for LPS, and 117 for Control.

Before pooling, 1 μg of each labeled sample was independently analyzed by MS to verify the completeness of the labeling. Strong cation exchange chromatography was performed as previously described [17] and samples were fractionated in a step-wide manner using the following concentrations of KCl (in 5 mM KH_2_PO_4_ and 25% acetonitrile, pH 2.9): 50, 100, 150, 200 and 350 mM. Samples were then desalted using C_18_ cartridges (Sep-Pak C18; Waters, Milford, MA, USA) following manufacturer’s instructions and finally dried under vacuum and stored at -20°C till LC-MS/MS analyses were performed.

### LC-MS/MS Analysis

All LC-MS/MS analyses were conducted with a LTQ-Orbitrap XL mass spectrometer (ThermoFisher Scientific, Waltham, MA, USA) coupled online with a nano-HPLC Ultimate 3000 (Dionex−ThermoFisher Scientific, Waltham, MA, USA) using a 10 cm picofrit column (I.D. 75 mm, New Objectives, Woburn, MA, USA) packed in house with C_18_ material (Aeris Peptide 3.6 mm XB-C18, Phenomenex). Chromatographic and instrumental conditions were as previously reported [18]. To increase protein identification and robustness of quantification, all peptides identified in each sample (as described in the next section) were used to generate a static exclusion list that was then used to perform (under the same chromatographic and instrumental conditions) a second LC-MS/MS run for each sample fraction.

### Data Analysis

Raw files were analyzed with a MudPIT protocol using Proteome Discoverer 1.4 (ThermoFisher Scientific, Waltham, MA, USA) connected to a Mascot server (version 2.2.4, Matrix Science, London, UK). MS/MS spectra were matched against the human section of the Uniprot database (version 20150107, 89706 entries), setting trypsin as digesting enzyme with up to one missed cleavage. Peptide and fragment tolerance were set to 20 ppm and 0.6 Da, respectively. Methylthiocysteine, 4-plex iTRAQ at the N-terminus and Lys were set as fixed modifications, while methionine oxidation was set as variable modification. A search against a randomized database and the algorithm Percolator were used to assess the false discovery rate (FDR). Data were filtered to keep only proteins identified with high confidence (q ≤ 0.01) and quantified with at least 2 unique peptides. iTRAQ ratios were normalized on the protein median value and data from both replicates were merged to obtain a single list of quantified proteins.

### Glutathione Analysis

Samples of colonic mucosa (0.2–0.4 g) were freshly put into 0.5% metaphosphoric acid in 1:2 ratio (w:v), minced with a surgical scissor at 4°C, frozen in liquid nitrogen and stored at -80°C for glutathione determination. Glutathione pool and redox state were measured as previously described [19].

### Ex Vivo Experiments on Human Colonic Smooth Muscle Strips

#### Isolation of smooth muscle strips

After removal of the mucosa and submucosa layers, colonic smooth muscle was cut into small strips (10-mm long by 2-mm wide, 0.10±0.05 g weight) by sharp dissection, identifying the circular orientation, according to the position of the taenia coli. The smooth muscle strips (SMSs) were mounted in separate 10-mL chambers as previously described [20]. Strips were initially stretched to 15 g of load, to bring them near conditions of optimum force development, and equilibrated for an additional 30 minutes after continuous perfusion with carbogenated Krebs solution. The solution was equilibrated with carbogen at pH 7.4 and at 37± 0.5°C.

#### Assessment of contractile activity of SMSs

During the perfusion period, spontaneous phasic contractions of SMSs developed gradually and stabilized after 30 minutes of equilibration. After stabilization, strips were stimulated with a maximally effective dose of acetylcholine (10^−5^ M Ach) in order to test the physiologic cholinergic response. After 10 minutes of washout, the experimental protocol was performed. The human colonic SMSs were exposed to N-undernatant or to LPS-undernatant or to LPS+INU-undernatant for 30 minutes and afterwards stimulated with a maximally effective dose of acetylcholine (10^−5^ M Ach). Isometric contractions were measured using force displacement transducers connected with a computer using MacLab system (Oxford, UK). In muscle strips experiments, the amplitude of the contractions was measured in grams. Ach-induced response was assessed calculating the percentage of maximal response compared to basal tone. Following supernatants exposure, Ach-induced contraction was compared to that obtained in basal condition.

### RNA Extraction and qPCR Analysis

Intestinal samples, taken from five individuals and exposed either to Krebs solution (control), LPS or LPS+INU were used for total RNA extraction. Samples were kept frozen and, after adding 1 mL of TRI Reagent (Sigma Aldrich, Milan, Italy) per every 100 mg of tissue, they were homogenized using T10 basic Ultra-Turrax (IKA, Staufen, Germany), working on ice. Subsequent steps were performed according to the manufacturer’s instructions. For real-time quantitative PCR (qPCR), cDNA was generated from 2 μg of RNA by using the iScript cDNA synthesis kit (Biorad, Milan, Italy). Gene expression analyses were performed in triplicate, either on single patients’ RNA or on a pool of their RNAs, using a CFX96 thermal cycler (Biorad, Milan, Italy) and the iTAQ Universal Sybr Green Supermix (Biorad, Milan, Italy). Relative mRNA quantification was obtained using the 2^-DDCt method, using the geometric average of the Cqs of three reference genes, namely beta-2-Microglobulin, GAPDH and HPRT for normalization purposes. Melting curve analysis was performed to ensure that single amplicons were obtained for each target. Primers for the genes under investigation were designed to have at least one of the primers in the pair designed on an exon-exon junction, or to encompass at least one intron, except for UGT2B4. For primer design and thermodynamic analysis of their quality the following programs were used: the Primer-Blast tool at NCBI (http://www.ncbi.nlm.nih.gov/tools/primer-blast/), OligoCalc (http://biotools.nubic.northwestern.edu/OligoCalc.html) and the IDT SciTools (http://eu.idtdna.com/pages/scitools). Primer sequences are reported in S1 Table.

### Statistical Analysis

Statistical analysis were performed on all experimental results by GraphPad Prism statistical software program 4.02 version by using different statistical analyses as indicated in the figures and tables. Data concerning glutathione measurements were subjected to one-way analysis of variance (ANOVA). For gene expression analyses, ANOVA was performed on the data obtained from the five individuals, considered as “biological replicates”. Differences were considered to be statistically significant with P <0.05. Student’s *t* test was used for statistical analysis of smooth muscle strips experiments. A P value < 0.05 was considered significant. For proteomic analysis, a two-tailed z-test was performed and only proteins that showed a iTRAQ ratios above 1.3 or below 0.7 and with p value ≤ 0.05 were considered as significantly altered.

## Results

### Proteomic Analysis

iTRAQ analysis led to the identification of 865 proteins within the human genome. iTRAQ ratios above at least 1.3 (1.3-fold induction and higher) were assumed to indicate up-regulation. The opposite scenario (values below 0.7) denoted down-regulation. A two-tailed z-test was used to select only proteins that showed a statistically significant (p <0.05) altered abundance. By employing this filter, we identified 24 differentially regulated proteins in LPS and LPS+INU samples compared to the control: among them 9 were differentially expressed only in LPS treated cells indicating that inulin exposure was able to restore the LPS-dependent alteration. Eight proteins were significantly altered only in LPS+INU treated cells (Table 1). We classified the differentially expressed proteins using PANTHER (protein analysis through evolutionary relationships) classification system to obtain an understanding of the molecular and functional characteristics.

**Table 1:** List of the proteins that showed significantly (p <0.05) altered abundance in LPS and LPS+INU samples in comparison with the control.

Uniprot accession number	Protein description	LPS/CTR	LPS+INU/CTR
**P62158**	Calmodulin OS = Homo sapiens GN = CALM1 PE = 1 SV = 2 - [CALM_HUMAN]	**1,8**	**1,0**
**Q5T7C4**	High mobility group protein B1 OS = Homo sapiens GN = HMGB1 PE = 1 SV = 1 - [Q5T7C4_HUMAN]	**0,6**	**1,0**
**Q15746-9**	Isoform 7 of Myosin light chain kinase, smooth muscle OS = Homo sapiens GN = MYLK—[MYLK_HUMAN]*	**0,6**	**1,0**
**P24844**	Myosin regulatory light polypeptide 9 OS = Homo sapiens GN = MYL9 PE = 1 SV = 4 - [MYL9_HUMAN]	**0,6**	**1,0**
**Q9Y2Q3**	Glutathione S-transferase kappa 1 OS = Homo sapiens GN = GSTK1 PE = 1 SV = 3 - [GSTK1_HUMAN]	**0,6**	**1,0**
**I3L397**	Eukaryotic translation initiation factor 5A-1 (Fragment) OS = Homo sapiens GN = EIF5A PE = 1 SV = 3 - [I3L397_HUMAN]	**0,5**	**1,0**
**E9PE82**	Short-chain-specific acyl-CoA dehydrogenase, mitochondrial OS = Homo sapiens GN = ACADS PE = 1 SV = 1 - [E9PE82_HUMAN]	**0,5**	**1,0**
**Q6P996-3**	Isoform 3 of Pyridoxal-dependent decarboxylase domain-containing protein 1 OS = Homo sapiens GN = PDXDC1 - [PDXD1_HUMAN]	**1,5**	**1,0**
**P27695**	DNA-(apurinic or apyrimidinic site) lyase OS = Homo sapiens GN = APEX1 PE = 1 SV = 2 - [APEX1_HUMAN]	**1,3**	**1,0**
**Q6ZN40**	Tropomyosin 1 (Alpha), isoform CRA_f OS = Homo sapiens GN = TPM1 PE = 1 SV = 1 - [Q6ZN40_HUMAN]	**0,6**	**0,6**
**P07951-2**	Isoform 2 of Tropomyosin beta chain OS = Homo sapiens GN = TPM2 - [TPM2_HUMAN]	**0,6**	**0,7**
**P21291**	Cysteine and glycine-rich protein 1 OS = Homo sapiens GN = CSRP1 PE = 1 SV = 3 - [CSRP1_HUMAN]	**0,6**	**0,6**
**P51911**	Calponin-1 OS = Homo sapiens GN = CNN1 PE = 1 SV = 2 - [CNN1_HUMAN]	**0,5**	**0,6**
**Q9NR12-2**	Isoform 2 of PDZ and LIM domain protein 7 OS = Homo sapiens GN = PDLIM7 - [PDLI7_HUMAN]	**0,5**	**0,7**
**P17661**	Desmin OS = Homo sapiens GN = DES PE = 1 SV = 3 - [DESM_HUMAN]	**0,7**	**0,6**
**Q99832-4**	Isoform 4 of T-complex protein 1 subunit eta OS = Homo sapiens GN = CCT7 - [TCPH_HUMAN]	**1,3**	**0,8**
**P09669**	Cytochrome c oxidase subunit 6C OS = Homo sapiens GN = COX6C PE = 1 SV = 2 - [COX6C_HUMAN]	**1,0**	**1,3**
**P02795**	Metallothionein-2 OS = Homo sapiens GN = MT2A PE = 1 SV = 1 - [MT2_HUMAN]	**1,0**	**1,3**
**P49748-2**	Isoform 2 of Very long-chain specific acyl-CoA dehydrogenase, mitochondrial OS = Homo sapiens GN = ACADVL—[ACADV_HUMAN]	**1,0**	**1,3**
**O75795**	UDP-glucuronosyltransferase 2B17 OS = Homo sapiens GN = UGT2B17 PE = 2 SV = 1 - [UDB17_HUMAN]	**1,0**	**1,7**
**P06133**	UDP-glucuronosyltransferase 2B4 OS = Homo sapiens GN = UGT2B4 PE = 1 SV = 2 - [UD2B4_HUMAN]	**1,0**	**1,5**
**Q01995**	Transgelin OS = Homo sapiens GN = TAGLN PE = 1 SV = 4 - [TAGL_HUMAN]	**1,0**	**0,7**
**A0A087WZF1**	Lipoma-preferred partner OS = Homo sapiens GN = LPP PE = 1 SV = 1 - [A0A087WZF1_HUMAN]	**1,0**	**0,7**
**H0YL34**	Synemin OS = Homo sapiens GN = SYNM PE = 1 SV = 1 - [H0YL34_HUMAN]	**1,0**	**0,6**

* MYLK is indicated with the alias MLCK in the text.

**Fig 1:**
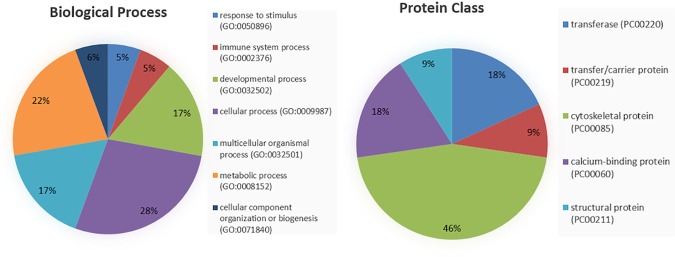
Functional distribution of the differentially regulated proteins in LPS and LPS+INU samples compared to the control, according to biological process and protein class.

In total, 7 biological processes and 5 protein classes were classified. The most important biological categories were cellular, metabolic, development processes and response to stimulus. The most important protein categories were cytoskeletal protein, calcium binding protein and transferase (Fig 1).

Inulin exposure was able to restore, in human colonic mucosa, the LPS-dependent alteration of some proteins involved in the host response and in the intestinal smooth muscle contraction such as the myosin light chain kinase (MLCK) and the myosin regulatory subunit 9 (MYL9) and to reduce the upregulation of two proteins involved in the radical-mediated oxidative stress induced by LPS (the DNA-apurinic or apyrimidinic site lyase) APEX1 and the T-complex protein 1 subunit eta CCT7). Moreover, the administration of inulin entailed a higher level of some detoxification enzymes (the metallothionein-2 MT2A, the glutathione–S-transferase K GSTk, and two UDP- glucuronosyltransferases UGT2B4, UGT2B17) compared to the LPS treatment.

**Fig 2:**
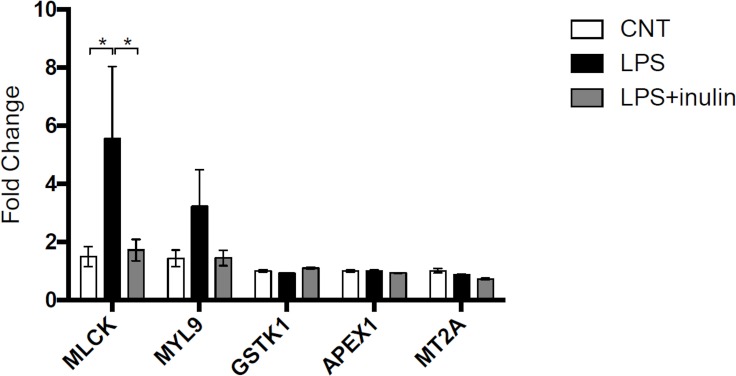
Changes in the levels of specific transcripts in response to LPS or LPS+INU treatments, compared to control condition. Values are means ± SE. The results were analyzed by ANOVA test. * indicates significant values (p < 0.05).

To investigate the mechanisms underlying the changes in protein levels, the levels of transcript corresponding to some of the differentially accumulated proteins were analyzed by qPCR. No differences in transcript levels were observed for most of the genes analysed, including GSTK1, APEX1 and MT2A (Fig 2). However, MLCK and MYL9 transcript levels were strongly increased after LPS treatment, whereas they remained unchanged in the LPS+INU condition (Fig 2).

### Human Smooth Muscle Strips Analysis

**Fig 3:**
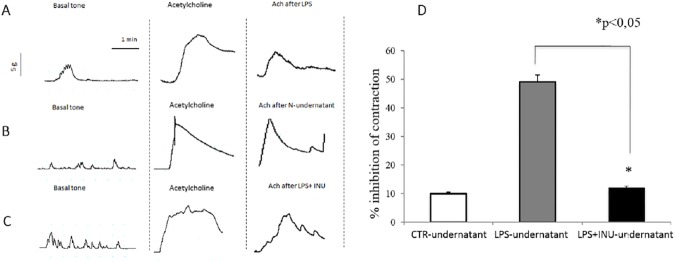
Selected representative traces showing colonic strip muscle activity after exposure to the LPS-undernatant, (A), to the N-undernatant (B, p<0.05.) and after pre-incubation of LPS with inulin (C, p = ns versus N-undernatant). Percentage of inhibition of contraction after undernatants administration (D). Values are means ± SEM of each group. The results were analyzed by Student’s t-test. *p < 0.05.

Colonic muscle strips, after one-hour of stabilization, developed basal tone and spontaneous activity, consisting of small amplitude rhythmic contractions of myogenic origin resented and a maximal contraction to Ach of 68.6± 8.5% above the baseline; after 10 min of washout, basal tone was restored. Following 30-min exposure to the LPS-undernatant, a significant decrease in maximal Ach-induced contraction was observed when compared to the contraction induced in CTR muscle strips incubated with the N-undernatant (49±5% vs 10±1% respectively, P<0.05) and this was completely prevented by the pre-incubation of the LPS with inulin (12±2%, P = ns versus N-undernatant) (Fig 3).

### Glutathione Pool

**Fig 4:**
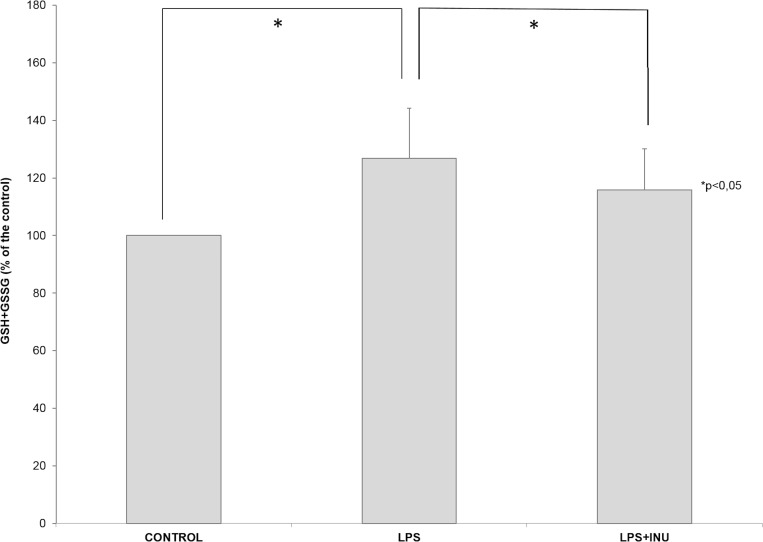
**(A)** The level of reduced glutathione (GSH) and (B) glutathione redox state (GSH/GSH+GSSG) in mucosa layer submitted to different experimental conditions (Control, LPS and LPS+Inulin treatments). The results were analyzed by ANOVA test. *p < 0.05.

GSH level increased in colonic mucosa after LPS treatment compared to the control. Whereas the presence of inulin during LPS treatment maintained GSH content of colonic mucosa at basal levels (Fig 4A). The levels of oxidized glutathione (GSSG) were very low and the glutathione redox state, calculated as reduced glutathione/reduced + oxidized glutathione ratio, did not change in colonic mucosa after LPS and LPS+inulin exposure compared to the control (Fig 4B). Our data suggest that glutathione might be involved in the colonocytes’ response to LPS exposure, a response blocked by the presence of inulin. Indeed the LPS-induced increase in GSH is blocked in the presence of inulin.

## Discussion

Data from the present study confirm the protective effect of inulin on the LPS induced colonic mucosal oxidative stress and muscle impairment. Using a iTRAQ analysis, we found that inulin was able to restore the level of some important protective proteins, involved in the inflammatory processes and was able to prevent impairment of smooth muscle contraction throughout the ability to revert the LPS-dependent alteration of some proteins involved in the intestinal smooth muscle contraction. We cannot exclude that the protective effects of inulin against LPS are mediated by a barrier effect that the fructose polymers might have interacting with LPS. However, proteomic analysis revealed that LPS+inulin treatment caused specific changes in the abundance of some proteins, different from those caused by LPS (Table 1). This evidence reinforces the hypothesis of a specific effect of inulin on colonic mucosa, the mechanism of which requires further investigation. Inulin-type fructans have been reported to stimulate saccharolysis in the colonic lumen and to favor the growth of lactobacilli and/or bifidobacteria [21]. The use of specific probiotics and prebiotics seems to be associated with reduced mucosal inflammation in experimental models of inflammatory bowel disease [22], but limited knowledge exists regarding the mechanism of action.

We have recently shown that inulin has an antioxidant activity that is not affected by treatments such as high temperature, pH changes and exposure to digestive enzymes [2], thus supporting the hypothesis that this fructan, besides modulating intestinal microbiota, can directly act on intestinal mucosa. Interestingly colonic mucosa exposure to inulin has been shown to prevent the LPS-induced impairment of the muscle contractility, possibly counteracting the mucosal production of free radicals as the amount of mucosal proteic carbonyl groups was reduced by about 60% compared to LPS exposed mucosa [2].

The existing literature on *in vivo* effect of inulin ingestion is limited. In clinical studies on human healthy volunteers administration of oligofructose and inulin promotes the growth of specific bacteria, especially Bifidobacteria and Lactobacillus, which have defined metabolic functions with health benefits, such as production of acetic and lactic acids and synthesis of B vitamins [1, 23–25]

Interestingly, in clinical studies on Inflammatory bowel disease (IBD) patients, prebiotics added to conventional therapy are able to obtain a clinical remission/response in 60% of patients [26]. Moreover in a small randomized controlled trial inulin was superior to placebo in decreasing endoscopic and microscopic inflammation in patients affected by pouchitis [27] even if the underlined mechanism in unknown. An interesting systematic review has shown that in adult patients with overweight or obesity administration of Inulin-type fructans is able to reduced serum levels of several inflammatory mediators, such as interleukin-6 and tumor necrosis factor, suggesting an anti-inflammatory action of this fiber [28].

In the present investigation, the effects of inulin on protection from inflammation was explored using a iTRAQ analysis. Interestingly, inulin was able to restore the level of some important proteins involved in protection from the radical-mediated oxidative stress induced by LPS, such as APEX1 and the T-complex protein 1 subunit eta CCT7. APEX1 is a multifunctional protein which plays a fundamental role in DNA repair and redox signaling by activating proteins involved in cellular response to several stresses such as inflammation and cancer. It has been shown that APEX1 controls the inflammatory response in LPS-stimulated macrophages [29] and it is significantly increased in the colonic epithelium of patients with ulcerative colitis [30]. In the present study, we also observed an increased content of the CCT subunit eta (CCT7) in LPS+INU treated samples compared to the control. CCT orchestrates the folding of many newly synthesized proteins and it is involved in membrane fusion events [31, 32]. Moreover, it seems to be important in the retrograde mitochondria signaling induced when mitochondria antioxidant defenses are overwhelmed. The excessive ROS production can trigger a mitochondrial unfolded protein response (UPRmt) involved in the field of mitochondrial dysfunction. It has been proposed that the UPRmt might thus represent one of the mechanisms that senses bacterial pathogens in the intestinal mucosae [33].

In LPS+inulin treated mucosa we also found the accumulation of the metallothionein MT2A. Metallothioneins, in addition to sequestering heavy metals, are also able to scavenge free radicals and to reduce damage from oxidative stress [34]. Our findings are in agreement with those of Inoue et al. [35] who demonstrated the anti-inflammatory effects of metallothioneins on disturbances of lung, kidney and liver induced by LPS. Moreover, metallothioneins are known to inhibit the NF-*k*B signaling [36] and to be downregulated in the intestinal mucosa of patients with inflammatory bowel disease and in gastrointestinal tumors [37, 38].

Another relevant finding from the present investigation is that, in LPS-treated mucosa, GSTk protein was down-regulated and inulin treatment was able to restore its level. GSTs are a superfamily of detoxification enzymes that play an important role in the protection of tissues against potentially harmful compounds [39]. In LPS+INU treated colonic mucosa not only the level of GSTk was restored but also two UGT enzymes were accumulated. The UGT gene family codes for 19 unique protein isoforms, which conjugate glucuronic acid to a wide range of both endogenous and xenobiotic substrates. The addition of the glucuronide to the target converts it into less bioactive compound and increases its water solubility promoting the excretion of the conjugate from the body. In this manner UGTs are able to detoxify a variety of well-known food carcinogens [40, 41]. In the intestinal mucosa of coeliac subjects, UGT activity is significantly lower than in the corresponding tissues of healthy people [42] and its expression decreases in colon cancer tissue as compared with normal tissues [43, 44]. Furthermore, variants of the UGT-enzyme family influence the risk to develop cancer after exposure to environmental and dietary carcinogens [44].

Analysis of transcript levels suggested that the observed changes of protein levels in LPS and LPS+INU treated cells were likely due to an altered protein turnover rather than to an altered regulation of mRNA levels.

Supporting our previous finding, demonstrating that inulin is able to restore colonic muscle cell contraction impairment due to LPS exposure [2], in the present investigation we found that the prebiotic restores the contractility of muscle strips in response to agonists. Impairment of contractility in gastrointestinal disorders is mainly due to an inflammatory process that starts in the intestinal mucosa and reaches the deeper muscle layer. In the present investigation we found that the administration of inulin not only entailed a higher level of detoxification enzymes and proteins involved in the radical-mediated oxidative stress, as already stated, but also reverted the LPS-dependent alteration of some proteins involved in the intestinal smooth muscle contraction. In particular, we found that MLCK and MYL9 alteration induced by LPS were counteracted by inulin exposure. MLCK is implicated in smooth muscle contraction via phosphorylation of MYL9 [45] and targeted deletion of MLCK in adult mouse smooth muscle resulted in severe gut dysmotility [46]. Nevertheless, the trend of expression of MLCK and MYL9, revealed by transcriptomic analysis, was contradictory. Whereas MLCK and MYL9 were among the downregulated proteins in LPS treated tissue, their mRNAs were increased, suggesting a possible regulative degradation at the protein level. The reduced net expression of MLCK by proteasome degradation has been reported in different tissues [47,48]. Even if these data are referred to the mucosal level of the enzymes, we cannot exclude that a similar mechanism could be present in the muscle layer (which include several cell types) and account, together with protection of muscle cells from oxidative stress, for the prevention of contractility impairment of the entire muscle strips. The rapid increase in MLCK transcript is in agreement with previous reports, showing that LPS treatment leads to a quick induction of MLCK mRNA in lung endothelial cells [49].

Finally, in our experimental system glutathione levels were specifically increased after LPS treatment, probably as an attempt to potentiate ROS or xenobiotic scavenging capacity. Interestingly, in LPS+INU glutathione remained at the control level. Supporting our findings, previous studies demonstrated a crucial role for glutathione in gastro-intestinal physiology and protection against pro-inflammatory conditions [50, 51]. Indeed, the impairment of glutathione detoxification activity toward ROS, such as the decreased activity of glutathione peroxidases 1and 2 in KO mice, led to severe colitis and colonic mucosa destruction [52,53].

In conclusion, our study demonstrates that inulin is able to modulate responses to pathogenic bacterial insults and to protect the colon from inflammatory processes probably by stimulating defense against ROS through the upregulation of colonic mucosal detoxification enzymes and proteins involved in the prevention of the radical-mediated oxidative stress. These data support the clinical beneficial effect of this fructan and highlight putative pathways involved in its protective function. Further investigations are needed to confirm these interesting data, obtained using an experimental model; as most of the ingested fructans would be fermented by the intestinal bacteria, clinical studies should be performed to support the existing literature from human cell cultures and animal studies regarding the anti-inflammatory properties of inulin.

## Supporting Information

S1 Table Primer sequences. For primer design and thermodynamic analysis of their quality the following programs were used: the Primer-Blast tool at NCBI (http://www.ncbi.nlm.nih.gov/tools/primer-blast/), OligoCalc (http://biotools.nubic.northwestern.edu/OligoCalc.html) and the IDT SciTools (http://eu.idtdna.com/pages/scitools).
(DOC)

## Abbreviations

LPS: lipopolysaccharide
Ach: acetylcholine
SMSs: smooth muscle strips
MLCK: myosin light chain kinase
MYL9: myosin regulatory subunit 9
APEX1: the DNA-(apurinic or apyrimidinic site) lyase
MT2A: the metallothionein-2
GSTk: the glutathione–S-transferase K
UGT: UDP- glucuronosyltransferases
ROS: reactive oxygen species
