# Supplementary material for: Effect of Inulin on Proteome Changes Induced by Pathogenic Lipopolysaccharide in Human Colon
Source: PLoS One. 2017 Jan 9;12(1):e0169481. doi: 10.1371/journal.pone.0169481 (PMC5222518; doi:10.1371/journal.pone.0169481)
Supplement: S1 Table — For primer design and thermodynamic analysis of their quality the following programs were used: the Primer-Blast tool at NCBI (http://www.ncbi.nlm.nih.gov/tools/primer-blast/), OligoCalc (http://biotools.nubic.northwestern.edu/OligoCalc.html) and the IDT SciTools (http://eu.idtdna.com/pages/scitools). (DOC) [file pone.0169481.s001.doc]

Table S1. Sequences of primers used for gene expression analysis in this study.

| **Gene** | **Ref_Seq** |  | **Sequence (5' --> 3')** | **Amplicon (bp)** |
| --- | --- | --- | --- | --- |
| **CALM1** | NM_006888.4 | Fw | CTCGCACCATGGCTGATCA | 125 |
|  |  | Rv | TGACCTCATGACAGTTCCAAGT |  |
| **APEX1 (variant 4)** | NM_001244249.1 | Fw | GGTCAGCTCCTTCGGACAA | 93 |
|  |  | Rv | CCTCATCGCCTATGCCGTAAG |  |
| **CCT7 (variant 4)** | NM_001166285.1 | Fw | TTTCTATTGCGCGAGGCATTG | 148 |
|  |  | Rv | TGTACTATGCTGCCTCTGCAC |  |
| **MYLK (variant 7)** | NM_053031.2 | Fw | GCAATGATCTCAGGGCTCAGT | 102 |
|  |  | Rv | GAAAGCTTGGGACACATCTTCAG |  |
| **MYL9 (variants 1-2)** | NM_006097.4; NM_181526.2 | Fw | CCCAGTTCCACGCACCC | 76 |
|  |  | Rv | ACATCTTGGCTTCTGGTGGG |  |
| **GSTK1**  **(variants 1-2-3-4)** | NM_015917.2; NM_001143679.1; NM_001143680.1; NM_001143681.1 | Fw | TGTCTTTCCCCTACCCCCAA | 91 |
|  |  | Rv | CTGAAAGGCACTTGTGAGGC |  |
| **MT2A** | NM_005953.3 | Fw | GACTCTAGCCGCCTCTTCAG | 141 |
|  |  | Rv | CAGGGCAGCAGGAGCAG |  |
| **UGT2B4**  **(variants 1-2-3)** | NM_021139.2; NM_001297615.1; NM_001297616.1 | Fw | CTTTGGATGTGACTGGGTTCCT | 140 |
|  |  | Rv | AGCTTCCAGCCTCAGACGTA |  |
| **PSMA6** | NM_001282232.1 | Fw | AGGGTCGGCTCTACCAAGTA | 104 |
|  |  | Rv | AAGGCCACCCTGGTTAATAGC |  |
| **HMGB1**  **(variants 1-2-3 etc)** | NM_001313893.1; NM_002128.5; NM_001313892.1 | Fw | ATCCCTCCCAAAGGGGAGAC | 84 |
|  |  | Rv | GCAGAAGAGGAAGAAGGCCG |  |
